# Personalized Approaches to Diagnostic and Therapeutic Strategies in Periprosthetic Fracture-Related Infections (PFRIs): Case Series and Literature Review

**DOI:** 10.3390/jpm15120576

**Published:** 2025-11-28

**Authors:** Marianna Faggiani, Marco Zugnoni, Matteo Olivero, Salvatore Risitano, Giuseppe Malizia, Silvia Scabini, Marcello Capella, Stefano Artiaco, Simone Sanfilippo, Alessandro Massè

**Affiliations:** 1 Department of Orthopedic Surgery and Traumatology, Trauma Center of Turin, “Città Della Salute e Della Scienza”, 10126 Turin, Italy; maolivero@cittadellasalute.to.it (M.O.); srisitano@cittadellasalute.to.it (S.R.); marcello.capella84@gmail.com (M.C.); stefano.artiaco@unito.it (S.A.); alessandro.masse@unito.it (A.M.); 2 Department of Orthopedics and Traumatology (Ortopedia e Traumatologia 1U), University of Turin, 10126 Turin, Italy; marco.zugnoni@unito.it (M.Z.); simone.sanfilippo@unito.it (S.S.); 3 Sports Science and Rehabilitation University of Turin, 10126 Turin, Italy; maliziabep@live.it; 4 Department of Medical Sciences, Infectious Diseases, University of Turin, 10126 Turin, Italy; sscabini@cittadellasalute.to.it

**Keywords:** periprosthetic fracture-related infection, fracture-related infection, orthopedic implant infection, infection, open reduction internal fixation, orthopedic complication, fracture

## Abstract

**Aim**: Periprosthetic fracture-related infections (PFRIs) are a serious complication of total arthroplasty, with incidence rates increasing in line with the growing number of joint replacements. PFRI can lead to prolonged hospitalization, multiple surgical procedures and suboptimal functional outcomes. The diagnosis of PFRI remains challenging due to the overlap of clinical symptoms with other post-traumatic conditions, and identification of the pathogen often fails through conventional methods. This study also highlights the importance of a personalized medicine approach in managing PFRI, where diagnostic and therapeutic decisions are tailored to the individual patient’s comorbidities, immune status and bone healing capacity. By integrating clinical, microbiological and imaging data, our findings support precision-based strategies to optimize outcomes and minimize complication. **Methods**: This retrospective case series was conducted at the Unit of Osteoarticular Infection of the University of Turin, Italy, from January 2018 to December 2023. Patients who developed septic complications after open reduction and internal fixation (ORIF) of periprosthetic fractures involving hip or knee implants were included. The infection was diagnosed in accordance with established guidelines, and treatment decisions were based on clinical, microbiological and radiological findings. **Results**: In the present study, periprosthetic fractures complicated by infections were identified in nine patients (5.4%), constituting a small but significant subset of cases. The cases were then categorized into four clinical scenarios based on the following variables: joint involvement, fracture healing and infection progression. Scenario A, involving fractures without prosthetic involvement and unhealed fractures, included three patients (33%) and was treated with debridement and change of the fixation device. Scenario B, involving fractures without prosthetic involvement but with healed fractures, involved one patient (11%), where the ongoing infection was confirmed despite the healed fracture and where the device could be removed. The third scenario (C), which pertains to cases involving prosthetic involvement, included three patients (33%) who required replacement or removal of the prosthesis and, in some cases, a second stage. The fourth scenario, involving patients with limited operability, included two patients (22%) for whom no surgery was performed. Despite the significant clinical challenges encountered, the paucity of literature on the management of periprosthetic fractures with septic complications is limited, highlighting the need for further research in this understudied area. **Conclusions**: PFRI remains a challenging complication that necessitates a multidisciplinary approach to diagnosis and treatment. Despite advances in imaging and microbiological testing, the early detection and identification of pathogens remain challenging, emphasizing the necessity for enhanced diagnostic methods. This study offers valuable insights into the management of PFRI and provides a foundation for future research to develop optimal diagnostic and therapeutic strategies.

## 1. Introduction

Periprosthetic fracture-related infection (PFRI) is a severe post-traumatic complication that typically occurs when an open internal osteosynthesis (ORIF) of a periprosthetic fracture becomes contaminated with pathogens, leading to an infection that may involve the bone, the fixation device and prosthetic components. It is associated with multiple surgeries, longer hospital stays and worse outcomes, jeopardizing the general health of patients [1].

The incidence of PFRI has risen over the past few years, ranging from 5% to 15% in periprosthetic fractures [2,3], and it is expected to increase due to the increase in the average age of the population and the increase in total joint replacements worldwide [1,2].

Fracture-related infections (FRIs) and periprosthetic joint infections (PJIs) have been well documented in the recent literature; however, PFRI remains a relatively under-researched area with limited studies, and the diagnosis and treatment are still unclear [4,5]. Furthermore, the clinical presentation in the early stage is confounding, and inflammatory signs such as redness, swelling and fever can be nonspecific and common in a variety of post-traumatic conditions leading to a difficult or delayed diagnosis of PFRI [6,7]. Without technical innovation, pathogen identification through standard culture can be difficult, leading to false negatives [7,8]. Imaging methods such as echography, computer tomography scan (CT), magnetic resonance imaging (MRI) and nuclear imaging are mostly used to diagnose bone and joint infection, but these techniques also have limitations in detecting PFRI due to the presence of artifacts from prosthetic components and synthetic devices [9,10].

Certainly, management of the PFRI is challenging, and it involves decisions regarding retention or removal of the associated synthesis device and retention or removal of the prosthesis [11,12]. The success of treatment depends on the timing of diagnosis, the surgical approach chosen and the host’s risk factors [13,14]. This study aims to analyze a case series of patients affected by PFRI, treated in a single orthopedic center and compared with existing literature in order to propose an effective diagnostic protocol and therapeutic strategies for managing PFRI [15]. In recent years, the management of complex orthopedic infections has progressively evolved toward the principles of personalized medicine. This paradigm emphasizes patient-specific assessment, integrating host factors, such as metabolic disorders, immune competence and microbiome variability, with microbial and imaging data to guide targeted interventions. Within this framework, PFRI management benefits from precision diagnostic tools and individualized treatment pathways that adapt to each patient’s biological and clinical profile.

## 2. Materials and Methods

A retrospective study was conducted on a series of patients treated in the Osteoarticular Infection Unit in the Orthopedic and Traumatological Centre, University of Turin, in Italy, from January 2018 to December 2023. All patients who underwent surgery for a periprosthetic fracture and developed infection associated with fixation devices were enrolled. The authors evaluated all patients over 18 years, with onset of septic symptoms following open reduction and internal fixation (ORIF) for periprosthetic fractures involving hip or knee prostheses. Exclusion criteria included the following: patients affected by bone or joint infection not surgically treated with internal fixation for periprosthetic fractures, incomplete data records or patients without at least one year of follow-up after the infection symptoms. The research was performed by searching for the following data from the electronic database:

- Demographic data: age, sex, BMI;
- Comorbidities and ASA score (American Society of Anesthesiologists score): diabetes mellitus (DM), chronic hepatitis (CH), rheumatoid arthritis (AR), chronic kidney disease (CKD), history of osteoporosis treatment, active cancer treatment and smoking history;
- Traumatological data: mechanism of injury (high and low energy trauma), type of peri-prosthetic fracture (Vancouver, Felix and Rorabeck classification), radiographic imaging, surgical treatment and timeline;
- Data related to infection: clinical, microbiological and laboratory results.

According to guidelines for diagnosing fracture-related infection (FRI) developed by the AO Foundation and the European Bone and Joint Infection Society [5], the authors recognized infection when at least one of the following confirmatory criteria was present: fistula or device exposition, purulent drainage, pathogen identification from at least two deep tissue cultures with phenotypic similarity. Suspicion of infection was raised when the following signs were present: clinical signs (redness, swelling, warmth or fever), elevated serum inflammatory markers (e.g., CRP, ESR, WBC) or imaging findings indicative of infection (e.g., echography, CT, MRI or PET scans) [9,10].

Furthermore, according to the PJI diagnosis guidelines [5], a prosthetic involvement of septic pathology was considered potential when at least two minor criteria were present (radiological signs/radiolucent lines, wound dehiscence, history of fever or bacteremia, clinical signs of loosening of the prosthesis components) or a major criterion (sinus tract or prosthetic exposure). For every patient, except for those ineligible for surgery, deep tissue samples were collected during surgery. At least five intraoperative samples were collected in the area of suspected PFRI. Tissue samples were collected from tissues at the level of the bone/hardware interface, whereas bone samples were collected from sequestra or loose bone fragments. If possible, bone marrow from the intramedullary space was collected.

Any removed hardware implant was sent to the laboratory for sonication. Each sample was collected using separate surgical instruments to avoid cross-contamination and placed in sterile containers [7,9]. Trained surgeons of the orthopedic infection unit performed all surgeries, and an antibiotic therapy was administered according to the infectious disease specialist’s consultation. All treatment decisions were individualized based on the patient’s comorbidities, fracture pattern, microbiological findings and infection severity. Antibiotic regimens were adjusted according to renal function, prior antimicrobial exposure and drug tolerance, in line with precision medicine principles aimed at maximizing efficacy and minimizing toxicity.

However, the diagnostic criteria already mentioned, while essential, do not fully capture the complexities of diagnosing PFRI; therefore, the current study aims to systematically document cases in order to analyze the diagnostic and therapeutic strategies employed. The research seeks to address essential questions about the diagnosis and management of PFRI, including diagnosis of prosthetic involvement in PFRI, decision-making regarding fixation devices (whether to clean, replace or remove them) and timing for replacing mobile prosthetic components (e.g., DAIR procedures or explantation).

This scope ensures a comprehensive understanding of current practices and potential improvements in the management of PFRI. Furthermore, findings were compared to existing literature to refine diagnostic and treatment approaches.

The analysis of the collected data is expected to identify the following four primary scenarios:

• Fracture-related infection in a periprosthetic fracture without prosthetic involvement with unhealed fracture.
• Fracture-related infection in a periprosthetic fracture without prosthetic involvement with healed fracture.
• Fracture-related infection in periprosthetic fracture with prosthetic involvement.
• Fracture-related infection in periprosthetic fracture in patients with limited operability.

These scenarios will guide the stratification of patients for data analysis and inform the evaluation of diagnostic and therapeutic strategies.

The study was conducted in accordance with the Declaration of Helsinki, and approval was obtained from the institutional review board of Città della salute e della Scienza di Torino (Application number 913.248; 00082/2024). As this was a retrospective study, informed consent was not required for the use of anonymized patient data. Descriptive statistics were used to summarize demographic and clinical characteristics. Continuous variables were reported as means, and categorical variables as frequencies and percentages.

## 3. Results

Here, 190 patients with peri-prosthetic hip and knee fractures were analyzed, and 13% of the sample did not have sufficient data for the evaluation required by our study. A total of 168 patients treated for periprosthetic fractures of the hip and knee were therefore included in the analysis. At the end, we were able to identify nine patients who fit our strict inclusion criteria. Of these, three were men and six were women (Table 1). The mean age was 77.8 years (range, 57–86 years) (Table 1). The comorbidities described in Table 1 show that the most prevalent comorbidity was osteoporosis (9/9), followed by diabetes mellitus (3/9). Only one of them was an active smoker at the time of the trauma. The average BMI (Body Mass Index) and ASA score were respectively 27.3 (range 22.0–30.4) and 2.56 (range 2–3). Comorbidities such as osteoporosis, diabetes and chronic kidney disease significantly influence both the risk of infection and fracture healing. Osteoporosis impairs bone quality, diabetes compromises immune response, increasing infection susceptibility and renal insufficiency affects wound healing and antibiotic metabolism, all of which may have impacted the outcomes observed in our study population. Additionally, 66% (6/9) of the patients had hip periprosthetic fractures, and the other cases had knee periprosthetic fractures (Table 2). The observed variability in comorbidities and biological conditions among patients underscores the importance of individualized management. These findings suggest that stratifying patients according to risk factors such as diabetes, osteoporosis and renal function could improve therapeutic outcomes through tailored diagnostic and treatment strategies.

***Fracture types:*** Fractures around the stem of a THA (Total Hip Arthroplasty) were classified according to the Vancouver classification, whereas fractures around the femoral and tibial components of a TKA (Total Knee Arthroplasty) were classified according to the Rorabeck and Felix classifications [15,16,17]. Most fractures were femoral (7 out of 9), followed by tibial (2 out of 9) (Table 2). Furthermore, 89% of patients had suffered low-energy trauma. In most cases, the prosthetic implant was a first implant (78%). Prior to the primary TJA (Total Joint Arthroplasty), only one patient had a history of multiple interventions. At the time of the periprosthetic fracture, four cases were treated with open reduction and internal fixation (ORIF) combined with prosthesis revision. The remaining cases, however, were managed exclusively through ORIF of the periprosthetic fracture. On average, the surgical time for the periprosthetic fracture management (whether involving ORIF alone or combined with prosthesis revision) was found to be 198.9 min (range 130–290 min).

**Septic complication**: The average time to the onset of suggestive or confirmatory criteria of FRI [5] of infection was found to be 304 days from the day of the periprosthetic fracture surgery (range 16–1551 days). In 2 cases, infection signs appeared in less than 30 days after surgery. According to the diagnostic criteria of FRI [5], in four patients, the infection was confirmed before surgery in the presence of visualization of the fixation device or the presence of a sinus tract. In another three cases, there were signs of suspected infection, such as wound dehiscence or local redness, radiographic signs of infection or history of systemic sepsis. In other cases (two patients), no clinical signs of infection were present except for a delay in bone healing or mobilization of the fixation device. In these patients, deep tissue samples collected during re-osteosynthesis surgery confirmed the ongoing infection. Microbiological isolates included *Corynebacterium striatum* in one patient and coagulase-negative *Staphylococci* in the other. In our case series, diagnostic methods for identifying infections in patients with periprosthetic fractures included a combination of imaging, microbiological tests and clinical assessments. Ultrasound (44%) was the first non-invasive tool used to detect fluid collections. MRI (11%) and CT (66.7%) were utilized to assess bone damage and deep infections. Arthrocentesis (11%) and eco-guided fluid aspiration (33%) were performed to obtain synovial fluid and fluid samples for microbiological cultures. Unfortunately, we were not able to identify the bacteria responsible for the infection prior to surgery. In three cases, intraoperative cultures were negative. In reference to the previously mentioned scenarios, we identified three cases (33%) of PFRI without joint involvement and without fracture healed (Scenario A), one case (11%) of PFRI without joint involvement and with fracture healed (Scenario B), three cases (33%) of PFRI with joint involvement (three of which with healed fracture) (Scenario C) and two cases (22%) of PFRI in a patient with limited operability (Scenario D) (Table 3).

***The cohort:*** In our cohort, three patients fell into Scenario A, and in two cases, there was no preoperative suspicion of septic non-union. These cases underwent revision of the open reduction and internal fixation (ORIF), one of which included autologous iliac crest bone grafting. The septic nature of the refracture was confirmed only retrospectively, following positive intraoperative culture results. In both cases, targeted antibiotic therapy was administered according to the susceptibility profiles of the isolated pathogens. The third patient included had undergone ORIF for a Felix type 3A fracture and subsequently required removal of the fixation hardware at partial fracture consolidation due to severe soft tissue compromise with device exposure, necessitating coverage with a gastrocnemius flap. From a rehabilitative standpoint, the patient was maintained in a non-weight-bearing status to protect the fracture site during the initial healing phase. Weight-bearing was only introduced after radiographic confirmation of callus formation, indicating early bone healing. A gradual weight-bearing protocol was then implemented and tailored to the progression of fracture consolidation. This approach ensured mechanical protection in the early stages, while later promoting functional recovery through progressively increased loading on the affected limb. A custom-molded brace was applied to protect the incompletely healed fracture. It is worth noting that the patient declined the option of external fixation, which limited the available strategies for maintaining alignment and stability during the healing process. Intraoperative cultures were negative, most likely due to empirical antibiotic therapy that the patient had initiated independently before the hardware removal procedure; empirical antibiotic treatment was therefore continued.

The only patient classified under Scenario B underwent removal of the fixation hardware following confirmation of periprosthetic fracture union, along with meticulous debridement and irrigation of the surgical site. Intraoperative biopsy samples were obtained for appropriate microbiological cultures, and subsequent antibiotic therapy was tailored according to the susceptibility profile of the isolated pathogens.

All cases classified as Scenario C in our series were late or delayed PFRIs, according to the conventional temporal classification of fracture-related infections (FRIs). In such cases, retention of the prosthetic components is generally not indicated. When the fracture was deemed radiographically consolidated, management consisted of hardware removal followed by a revision procedure. Specifically, a one-stage revision was performed in one case with fracture union, whereas in the only case without fracture consolidation, a two-stage procedure was undertaken. In the latter, persistence of the infectious process necessitated an additional interim exchange of the antibiotic spacer before proceeding to the second stage.

In one case of prosthetic involvement with a healed fracture, it was necessary to remove not only the fixation hardware but also the prosthetic components. In this instance, the patient requested to postpone the revision procedure and subsequently failed to attend follow-up appointments. Two years later, she returned with severe femoral stem loosening and consequent substantial bone stock loss. Given the extent of bone deficiency, her limited functional demands and multiple medical comorbidities, a Girdlestone resection arthroplasty was performed. In all cases treated surgically, at least five deep bacteriological samples were taken and the unstable or broken devices were removed. In 2 cases, the patient did not undergo any surgical treatment from the outset due to their clinical condition (Table 3).

## 4. Discussion

Our case series identified nine patients with periprosthetic fracture-related infection (PFRI) among 168 individuals treated for periprosthetic fractures of the hip or knee, corresponding to a prevalence of 5.3%. This rate is relatively low compared to that reported in previous studies (5–15%) (1). The majority of affected patients were over 75 years old (mean age 77.8 years) and presented common comorbidities, such as osteoporosis and diabetes mellitus, both recognized as factors that negatively influence postoperative outcomes [13,14]. The mean time to onset of suspected or confirmed PFRI was 304 days, in line with previous findings [7]. Early diagnosis proved challenging due to the frequent overlap of clinical signs with other post-traumatic complications. Advanced imaging modalities, including MRI and CT, although widely employed, were often limited by artefacts from prosthetic components [9]. Microbiological testing was essential for confirmation; however, in several cases, preoperative samples failed to isolate the causative pathogen. Indeed, no infection in our cohort was microbiologically confirmed before surgery, underscoring the current limitations of available diagnostic tools. This finding aligns with the observations of [8] regarding the difficulty of detecting pathogens in bone and joint infections in the absence of overt clinical signs. In our series, diagnosis was based on a combination of imaging (ultrasonography 44%, MRI 11%, CT 67%), microbiology and clinical features, with ultrasound-guided sampling recommended to improve preoperative pathogen identification.

Management strategies varied according to the timing of diagnosis, the stage of fracture healing, and the presence or absence of prosthetic involvement, the latter representing a key determinant of the therapeutic approach. In cases without prosthetic involvement (Scenarios A and B), the clinical and therapeutic considerations closely resembled those of fracture-related infections (FRIs), where the priority is fracture consolidation followed by eradication or suppression of infection [18]. In Scenario A, where the fracture had not healed radiologically or fixation was unstable, device removal, local debridement and re-synthesis with possible biological grafting yielded favorable outcomes. In Scenario B, characterized by fracture healing and absence of prosthetic involvement, hardware removal and surgical debridement were effective in eradicating infection.

When prosthetic components were involved (Scenario C), management became more complex, requiring the integration of FRI treatment principles with those applied to periprosthetic joint infection (PJI). Accurate preoperative identification of prosthetic involvement was critical, as intraoperative assessment was technically challenging and not always conclusive. Advanced imaging, including CT and MRI, could demonstrate peri-prosthetic collections or other signs of infection, while radiolabeled leukocyte scintigraphy offered additional diagnostic value in orthopedic infections. However, the accuracy of scintigraphy may be reduced when the fracture is unhealed or within the first postoperative year due to the risk of false positives. Comprehensive imaging evaluation was therefore essential to guide therapeutic decisions and optimize outcomes. For Scenario C, surgical planning—whether to retain, revise or explant the prosthesis—depended on infection severity, fracture healing and patient condition. Early revision with removal of mobile components is to be preferred in acute infections, whereas chronic infections (>30 days) require prosthesis explanation for adequate infection control [15]. All surgical interventions were combined with targeted antibiotic therapy, based on intraoperative deep cultures, and, when indicated, soft tissue coverage with flaps.

**Figure 1:**
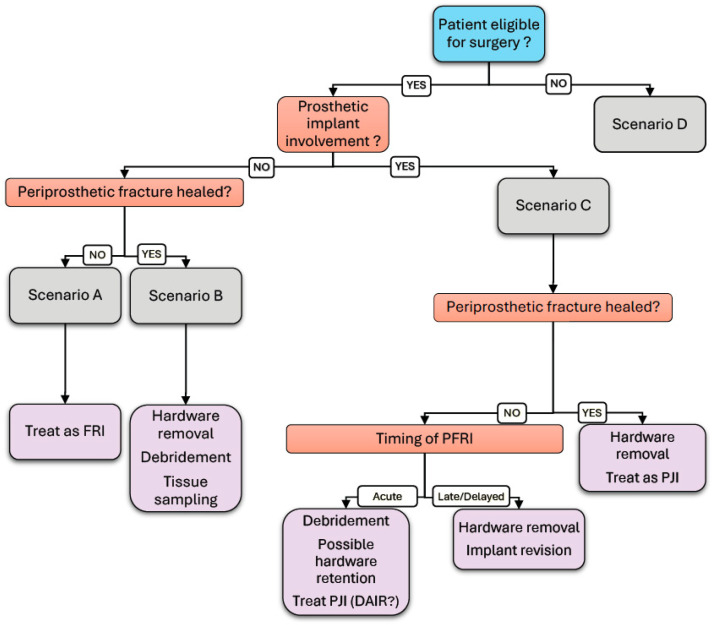
Diagnostic and surgical strategy workflow for periprosthetic fracture-related infection (PFRI). This flowchart guides the management of patients with suspected periprosthetic fracture-related infections (PFRIs), outlining decision points based on surgical eligibility, implant involvement, fracture healing and timing of infection to determine appropriate treatment strategies. It categorizes cases into scenarios A–D, recommending interventions, such as hardware removal, debridement or treatment as fracture-related infection (FRI) or periprosthetic joint infection (PJI).

This study supports the value of a standardized diagnostic workflow integrating imaging (Figure 1), microbiology and clinical evaluation to determine the optimal surgical strategy. Notably, in two cases, deep tissue cultures obtained during re-osteosynthesis revealed ongoing infection despite the absence of clinical signs, highlighting the need to consider infection in any delayed fracture healing, even in the absence of overt symptoms. The retrospective design and small sample size (nine patients) limit the generalizability of these findings, while the exclusion of patients without surgical fixation or with incomplete follow-up may have introduced selection bias. Nonetheless, this study constitutes the largest case series of periprosthetic fracture-related infection (PFRI) reported in the literature to date, and the results provide relevant insights into the diagnostic and therapeutic complexity of PFRI and establish a basis for future research. From a personalized medicine perspective, the management of PFRI should integrate multidimensional clinical, radiological and microbiological patient data to design individualized treatment algorithms. Precision diagnostic approaches, such as molecular pathogen detection and host-response biomarkers, may enhance early recognition of infection and guide targeted therapy. Incorporating patient-specific variables, including immune status and bone healing potential, could help predict prognosis and optimize surgical and pharmacologic strategies.

PFRI represents a particularly demanding clinical scenario due to the interplay between trauma, orthopedic implants and microbial contamination. The most frequent pathogens include *Staphylococcus aureus* (including MRSA), coagulase-negative staphylococci, and, less commonly, *Enterococcus* spp., *Corynebacterium* spp. and Gram-negative bacilli. *Corynebacterium striatum*, historically regarded as a skin commensal, has increasingly been recognized as a true pathogen in prosthetic infections, particularly when implant loosening is the sole clinical finding. Given the frequent absence of purulence and the difficulty in distinguishing contamination from true infection, especially intraoperatively, empiric antimicrobial therapy should provide broad coverage. Initial regimens typically combine vancomycin with a broad-spectrum beta-lactam or carbapenem, tailored according to local resistance patterns and individual patient factors. Antibiotic therapy is generally administered intravenously for an initial duration of 2 to 6 weeks, depending on the severity of infection, microbial findings and surgical strategy (e.g., debridement vs. implant removal). This is often followed by an oral step-down therapy for an additional 4 to 8 weeks. The total duration typically ranges from 6 to 12 weeks. The choice of oral agents is guided by culture and sensitivity results, with options including linezolid, fluoroquinolones or combination regimens with rifampicin for biofilm-active coverage. Culture-guided therapy remains essential to maximize treatment efficacy and reduce the risk of antimicrobial resistance.

## 5. Conclusions

Despite the small sample size and the resulting lack of statistically significant data, some relevant trends emerged. In cases of periprosthetic infection without joint involvement, it appears beneficial to remove the fixation hardware once the fracture has healed. If healing has not occurred, a revision osteosynthesis should be considered. In cases with joint involvement, either the exchange of mobile components or complete implant removal should be performed, depending on the infection’s presentation. Particular attention should be given to confirming, or definitively excluding, involvement of the prosthetic components by the infection. Empiric antibiotic therapy is essential in the early phase, followed by targeted treatment based on correctly obtained intraoperative cultures. These findings support the importance of individualized, scenario-based strategies within a multidisciplinary approach. Overall, our findings reinforce the value of a personalized scenario-based approach to PFRI management. Integrating patient comorbidities, microbiological profiles and imaging data into a unified framework supports the principles of precision orthopedic infection care.

## Figures and Tables

**Table 1:** Study population: the demographic and clinical characteristics of the study population, including age, gender, BMI, comorbidities and lifestyle factors.

Patient	Age (Year)	Gender	BMI	ASA	Smoking Status	Diabetes	Chronic Hepatitis	Rheumatologic Disease	Chronic Kidney Disease	Malignancy	Osteoporosis
1	78	M	22.00	3	1	1	0	0	1	0	1
2	84	F	23.47	2	0	0	0	0	0	0	1
3	86	F	27.34	3	0	0	0	0	0	0	1
4	74	M	30.25	3	0	1	0	0	0	0	1
5	57	F	33.76	2	0	0	0	0	0	0	1
6	84	F	25.39	2	0	0	0	0	0	0	1
7	85	M	23.18	3	0	0	0	0	0	0	1
8	78	F	30.42	2	0	1	0	0	0	0	1
9	81	F	29.30	3	0	0	0	0	0	0	1

Legend: 0 = no, 1 = yes.

**Table 2:** Periprosthetic fracture classification and treatment. Details the classification of periprosthetic fractures, the type of implant, surgical procedures performed and their durations.

Patient	Mechanism of Injury	Fracture Classification	First Implant	Surgical Procedure	Duration (Minutes)
1	HIGH ENERGY	Felix 3A	1	ORIF	165
2	LOW ENERGY	Vancouver B1	1	ORIF	130
3	LOW ENERGY	Vancouver B1	1	REVISION + ORIF	270
4	LOW ENERGY	Rorabeck 2	0	ORIF	210
5	LOW ENERGY	Rorabeck 2	1	REVISION + ORIF	290
6	LOW ENERGY	Vancouver B2	1	ORIF	140
7	LOW ENERGY	Vancouver C	1	ORIF	150
8	LOW ENERGY	Vancouver B3	1	REVISION + ORIF	240
9	LOW ENERGY	Vancouver B2	0	REVISION + ORIF	195

Legend: 0 = no, 1 = yes.

**Table 3:** Time 1: septic complication, diagnostic workup.

	Days Since Surgery	Local Signs of Infection					Preoperative Imaging			Scenario
Patient		Non-Union/Hardware Mobilisation	Local Wound Inflammatory Signs	Wound Dehiscence	Fistula	Hardware Exposition	Ultrasound	CT Scan	MRI	
1	62	0	1	1	0	1	0	0	0	B
2	249	0	1	0	1	0	1	1	0	C
3	1272	0	0	0	1	0	1	1	0	D
4	20	0	1	1	0	0	1	1	1	C
5	230	1	0	0	0	0	0	1	0	A
6	262	0	1	0	0	0	1	0	0	C
7	101	1	0	0	0	0	0	1	0	A
8	16	0	0	1	0	0	0	1	0	B
9	1551	0	1	0	1	0	0	0	0	C

Legend: 0 = no, 1 = yes.

## Data Availability

The raw data supporting the conclusions of this article will be made available by the authors on request.
